# Outcomes of Postoperative General Surgery Patients Requiring Mechanical Ventilation: A Prospective Observational Study

**DOI:** 10.7759/cureus.95479

**Published:** 2025-10-26

**Authors:** Priyathosh Lokesh K, Karthik N, Mohit Rakhecha, Indra Singh Choudhary, Mayank Badkur, Mahendra Lodha, Niladri Banerjee, Nikhil Kothari, Naveen Dutt, Ashok Puranik

**Affiliations:** 1 Department of General Surgery, AII India Institute of Medical Sciences, Jodhpur, Jodhpur, IND; 2 Department of General Surgery, All India Institute of Medical Sciences, Jodhpur, Jodhpur, IND; 3 Department of Anesthesiology and Critical Care, All India Institute of Medical Sciences, Jodhpur, Jodhpur, IND; 4 Department of Pulmonary and Critical Care Medicine, All India Institute of Medical Sciences, Jodhpur, Jodhpur, IND; 5 Department of Surgery, AIl India Institute of Medical Sciences, Guwahati, Guwahati, IND

**Keywords:** 30-day mortality, intensive care unit, laparotomy, mechanical ventilation, postoperative outcomes

## Abstract

Background: By identifying the risk factors that contribute to complications and difficulties in postoperative extubation, this study provides insights that can refine ICU strategies, guide clinical decisions, and ultimately enhance outcomes for surgical patients on mechanical ventilation. This study aimed to evaluate the postoperative outcomes of patients requiring mechanical ventilation in general surgery.

Methods: This single-centre, hospital-based, prospective observational study included elective and emergency general surgery postoperative patients aged 18 years or older who required postoperative mechanical ventilation. The collected data included demographics, American Society of Anaesthesiologists scores (ASA), quick Sepsis-Related Organ Failure Assessment scores (qSOFA), Acute Physiology and Chronic Health Evaluation II scores (APACHE II), procalcitonin trends, and postoperative complications. The primary objective was to describe outcomes in patients requiring mechanical ventilation, with a focus on 30-day in-hospital mortality.

Results: A total of 92 patients were enrolled during the study period, with 89 admitted to the ICU following emergency surgery and three after elective surgery. Within 30 days of surgery, mortality occurred in 59 patients, while 33 survived. The incidence of ventilator-associated pneumonia and multiple organ dysfunction syndrome was significantly greater among non-survivors than among survivors. Both qSOFA ≥ 2 and ASA > 3 scores were significantly associated with ventilator-associated pneumonia, multi-organ dysfunction syndrome, and 30-day mortality.

Conclusion: Our study underscores the importance of clinical biomarkers and scoring systems, including qSOFA, APACHE II, international normalised ratio (INR), albumin, and procalcitonin in predicting mortality and guiding management strategies for general surgery patients requiring mechanical ventilation.

## Introduction

Around 234 million major surgeries are performed annually globally, with associated mortality rates ranging from 0.4% to 4%. These rates can rise significantly in high-risk surgical patients, reaching 12.3% to 25% [1, 2]. ICUs are critical in managing high-risk patients and providing advanced monitoring and life-saving interventions. As a result, postoperative admission to the ICU is often essential for patients requiring close observation and mechanical ventilation.

The demand for postoperative intensive care frequently exceeds available resources, particularly in low- and middle-income countries such as India. Limited ICU capacity poses challenges in managing critically ill surgical patients, emphasising the need to identify high-risk individuals who may benefit most from postoperative mechanical ventilation and intensive monitoring [3, 4].

Recognizing patients at high risk for surgical complications and developing effective strategies to reduce morbidity and mortality represent significant challenges for anaesthesiologists and surgeons. Despite the high burden of postoperative ICU admissions in India, there is a paucity of systematic studies evaluating patient outcomes in this setting. The current literature on surgical ICU outcomes, being largely retrospective, provides limited insight into the complex factors influencing postoperative morbidity and mortality. This prospective observational study aims to bridge this gap by evaluating postoperative complications and mortality among general surgery patients admitted to the ICU. It seeks to identify key risk factors influencing complications and challenges in postoperative extubation. By understanding these factors, the findings can help optimize ICU strategies, improve decision-making, and enhance clinical outcomes for surgical patients requiring mechanical ventilation. This study aimed to evaluate postoperative outcomes in general surgery patients requiring mechanical ventilation, with the primary objective of assessing 30-day in-hospital mortality.

## Materials and methods

Study design and participants

This prospective, single-centre, hospital-based observational study was carried out in the Department of General Surgery and Intensive Care Unit at the All India Institute of Medical Sciences (AIIMS), Jodhpur, India, a tertiary care centre, between July 2021 and December 2022. The Institutional Ethics Committee approved the study (approval number: IEC/2021/3610). Eligible participants included elective and emergency general surgery postoperative patients aged 18 years or older who required postoperative mechanical ventilation. Exclusion criteria were patients undergoing surgery for head or chest trauma, those with preoperative ICU admission for any condition, COVID-19-positive status, and those extubated after the surgery but requiring reintubation later in the ward.

Study procedure and outcomes

All eligible postoperative patients admitted to the ICU underwent a comprehensive assessment as part of standard clinical practice. Detailed information regarding the study was provided to patient attendants through a patient information sheet, and written informed consent was obtained before enrolment. Documenting eligible participants' medical history, examinations, investigations, and scores was involved. Intraoperative details, including procedure, duration of surgery, haemodynamic parameters, blood transfusion, ICU factors, postoperative complications, and outcomes, were recorded. Data collected included demographics, American Society of Anaesthesiologists (ASA) scores, quick Sepsis-Related Organ Failure Assessment (q-SOFA) scores, Acute Physiology and Chronic Health Evaluation II (APACHE II) scores, operative duration, length of postoperative ICU stay, duration of intubation, blood transfusion requirements, use of total parenteral nutrition, vasopressor requirements, procalcitonin trends, culture results, complete blood count and postoperative complications. The primary objective was to identify outcomes in patients requiring mechanical ventilation for more than 48 hours, focusing on 30-day in-hospital mortality. Secondary objectives included identifying its associated risk factors for mortality and determining the causes of postoperative extubation failure. All collected clinical parameters were systematically analysed to achieve the study objectives. Patients were followed up until discharge, death, or 30 days postoperatively, whichever occurred first.

Sample size

This time-bound study included all general surgery postoperative patients who met the eligibility criteria. A formal a priori sample size calculation was not performed due to the exploratory nature of the study.

Statistical analysis

Data were compiled and analyzed using the IBM SPSS Statistics software, version 29 (IBM Corp., Armonk, NY). Descriptive statistics were presented as percentages, mean, and median, as appropriate. Comparative analyses were conducted using the Pearson chi-square test, Fisher’s exact test, independent t-test, and Mann-Whitney U-test. A p-value < 0.05 was considered statistically significant.

## Results

A total of 92 patients were enrolled during the study period, with 89 admitted to the ICU following emergency surgery and three after elective surgery. The median age among survivors was 60 years (IQR: 45-67 years), while that among non-survivors was 57 years (IQR: 40-68 years), with 63 males (68.5%) and 29 females (31.5 %). The baseline characteristics of the study participants categorised by survival status are detailed in Table 1. Within 30 days of surgery, mortality occurred in 59 patients, while 33 survived. The comparison of various preoperative laboratory parameters and clinical scoring systems associated with 30-day in-hospital mortality is detailed in Table 2.

**Table 1:** Baseline Characteristics of Patients Categorized by Survival Status SMA: superior mesenteric artery

Characteristic	Survivors (n = 33)	Non-survivors (n = 59)
Median age in years (IQR)	60 (45–67)	57 (40–68)
Sex (%)		
Female	13 (39.4%)	16 (27.1%)
Male	20 (60.6%)	43 (72.9%)
Median operative duration in minutes (IQR)	180 (160–195)	180 (165–225)
Comorbidities (%)		
Diabetes mellitus	09 (27.2%)	22 (37.3%)
Hypertension	07 (21.2%)	17 (28.8%)
Obstructive airway disease	04 (12.1%)	10 (17.0%)
Coronary artery disease	00 (0%)	05 (8.5%)
Chronic kidney disease	02 (6.0%)	03 (5.1%)
Opioid dependence (%)	09 (27.3%)	22 (37.3%)
Median Charlson Comorbidity Index (IQR)	2 (2–4)	2 (1–4)
Type of surgery (%)		
Emergency	31 (93.9%)	58 (98.3%)
Elective	02 (6.1%)	01 (1.7%)
Diagnosis (%)		
Perforation peritonitis	14 (42.4%)	29 (49.2%)
Acute intestinal obstruction	12 (36.4%)	09 (15.3%)
SMA thrombosis with bowel gangrene	02 (6.1%)	12 (20.3%)
Miscellaneous	05 (15.1%)	09 (15.2%)
Operative procedure (%)		
Exploratory laparotomy	28 (84.8%)	50 (84.7%)
Miscellaneous	05 (15.2%)	09 (15.3%)

**Table 2:** Comparison of Preoperative Clinical and Laboratory Parameters with 30-day Mortality *p-value calculated by Mann-Whitney U test qSOFA: quick Sepsis-Related Organ Failure Assessment; TLC: total leucocyte count; INR: international normalized ratio; APACHE II: Acute Physiology and Chronic Health Evaluation II

Characteristic	Non-survivors (59)	Survivors (33)	p-value*
	Median (IQR)		
qSOFA	1 (1 - 1)	2 (2 - 2)	<0.001
Preoperative hemoglobin (g/dL)	12 (9.9 - 13.7)	11 (9.1 - 12.6)	0.120
Preoperative TLC (×10³/cu mm)	10.5(6.3 - 18.1)	10.5 (6.4 - 21.6)	0.471
Preoperative platelets (×10⁵/cu mm)	2.9 (1.7 - 3.7)	2.0 (1.3 - 3.1)	0.025
Preoperative potassium (mmol/L)	4.4 (3.5 - 4.6)	4.2 (3.7 - 5.1)	0.754
Preoperative sodium (mmol/L)	134 (131 - 137)	133 (128 - 138)	0.579
Preoperative albumin (g/dL)	2.8 (2.5 - 3.5)	2.4 (1.9 - 2.9)	0.001
Preoperative creatinine (mg/dL)	1.3 (0.9 - 2.0)	1.4 (0.9 - 2)	0.832
Preoperative INR	1.4 (1.1 - 1.6)	1.5 (1.3 - 1.8)	0.030
APACHE II score at admission	21 (17 - 22)	24 (20 - 28)	<0.001
Procalcitonin day 1 (ng/mL)	12.5 (3.3 - 22.3)	25.6(13.5 - 49.4)	0.005
Procalcitonin day 3 (ng/mL)	3.7 (1.7 - 15.3)	33 (3.4 - 78.3)	0.002
Procalcitonin day 7 (ng/mL)	1.6 (0.3 - 3.5)	27.9 (5.6 - 80.6)	<0.001
Procalcitonin day 14 (ng/mL)	0.5 (0 - 4.6)	24.8 (7.9 - 100)	0.001
Procalcitonin day 21 (ng/mL)	0 (0 - 0)	55.8 (11.5 - 100)	0.071
Procalcitonin day 30 (ng/mL)	0 (0 - 0)	100 (100 - 100)	0.5

Postoperatively, 30.4% received blood transfusions, 16.3% underwent tracheostomy, and 15.2% required total parenteral nutrition. Non-survivors had significantly higher rates of ventilator-associated pneumonia and multi-organ dysfunction syndrome compared to survivors. Although surgical site infection rates were comparable between survivors and non-survivors, ventilator-associated pneumonia and multiple organ dysfunction syndrome demonstrated a strong association with mortality. A comparison of postoperative complications between survivors and non-survivors is shown in Table 3.

**Table 3:** Comparison of Postoperative Complications Between Survivors and Non-survivors *p-value calculated by chi-square test; **p-value calculated by Fisher’s exact test

Frequency of Complication	Survivors (n= 33)	Non-survivors (n= 59)	p-value
Surgical site infection	13	15	0.162*
Ventilator-associated pneumonia	03	20	0.008**
Multi-organ dysfunction syndrome	03	56	0.0001**

Both qSOFA ≥ 2 and ASA > 3 scores were significantly associated with ventilator-associated pneumonia, multi-organ dysfunction syndrome, and 30-day mortality. There was no significant correlation between the scores and surgical site infections. Table 4 shows a correlation of qSOFA and ASA scores with postoperative outcomes.

**Table 4:** Correlation of qSOFA and ASA Scores with Postoperative Outcomes *p-value calculated by chi-square test qSOFA: quick Sepsis-Related Organ Failure Assessment; ASA: American Society of Anaesthesiologists

Postoperative Outcome	qSOFA ≥ 2 (n=92)	ASA >3 (n=92)
Surgical site infection	16 (p = 0.920)	16 (p = 0.823)
Ventilator-associated pneumonia	19 (p = 0.03)	19 (p = 0.02)
Multi-organ dysfunction syndrome	45 (p = 0.0001)	44 (p = 0.0001)
30-day mortality	46 (p < 0.001)	45 (p < 0.0001)

## Discussion

Higher mortality rates were noted in males and patients aged 60 years or older, consistent with findings from a similar study conducted by Patel et al. [3]. The qSOFA score, initially validated in medical contexts, shows promise in both medical and surgical settings due to its components' consistent prognostic value [5]. Developed through data-driven methods, qSOFA has proven more accurate than the Systemic Inflammatory Response Syndrome (SIRS) score in predicting mortality and ICU transfers in suspected sepsis cases, despite concerns over reduced sensitivity impacting timely treatment initiation. The qSOFA score aids resource allocation but isn't definitive for organ dysfunction. It should prompt further investigation, treatment adjustments, and ICU consideration if needed. It should be used as part of a comprehensive clinical assessment rather than as a standalone decision-making tool. Our study links qSOFA ≥2 with higher 30-day mortality, ventilator-associated pneumonia, and multiple organ dysfunction syndrome risks, suggesting it as a threshold to identify high-risk patients for resource allocation in sepsis cases, similar to other results [6-8]. Our study did not identify a correlation between the qSOFA score and the occurrence of surgical site infections, primarily because surgical site infections are often superficial and do not manifest as systemic organ dysfunction, which the qSOFA score was designed to assess [9].

Consistent with previous studies demonstrating a higher ICU mortality risk in patients with ASA scores > 3 compared to those with lower ASA scores, our study findings align with this [1]. Monitoring and appropriately managing these high-risk patients are crucial to improving survival rates in the ICU. Unlike other research findings, our study did not observe a significant association between preoperative anaemia and mortality, contrasting with prior reports [10]. Serum albumin is essential in maintaining colloid oncotic pressures, acting as a carrier, and participating in metabolism and antioxidant functions. Hypoalbuminemia is associated with impaired physiological functions, including compromised immune responses and increased susceptibility to infections, which can contribute to higher mortality rates [11]. In ICU patients, low albumin levels predict longer stays, higher mortality, and increased readmissions due to poor health and immune function [12]. Our study found a significant correlation between serum albumin levels and the occurrence of mortality. However, unlike other studies, we did not find a significant link between the specific cutoff and the prediction of mortality or complications, possibly due to the smaller size of our study population [13]. Procalcitonin is a biomarker used in clinical practice to assess the severity of bacterial infections, particularly sepsis. Studies reveal that higher procalcitonin levels predict increased mortality risk within 28 days [14]. This underscores the role of procalcitonin as a prognostic indicator in ICU settings, suggesting that patients with elevated procalcitonin may require more intensive management and vigilant monitoring to improve their chances of recovery. Our study confirmed a significant relationship between procalcitonin levels and patient outcomes, consistent with findings from a systematic review [15].

Similar to other studies, the duration of anaesthesia did not show statistical significance in predicting mortality [3]. International normalised ratio (INR) measurements upon admission are independent predictors of mortality in critically ill patients, consistent with other studies, as indicated by a p-value of 0.030 [16]. These results underscore the utility of INR as a prognostic indicator in assessing patient outcomes in ICU settings, indicating coagulopathy or sepsis-related organ failure. In our study, platelet count upon admission reliably predicted mortality, aligning with the findings of a retrospective study by Hua et al. [17]. The APACHE II score is widely used in ICU settings to predict mortality and guide treatment decisions by assessing various physiological parameters and disease severity. Bahtouee et al.'s retrospective study demonstrated its effective predictive capability for overall mortality. Similarly, our study identified a correlation between the APACHE II score and mortality, emphasising its role in clinical decision-making to manage critically ill patients effectively [18]. Ejiro et al. reported an overall mortality rate of 28.4%, highlighting significant variability in mortality across different settings and patient groups [19]. Another study by Zhang et al. reported a higher mortality rate of 32.6%, differing from our findings [20]. Our patient group's specific characteristics, conditions, and variations in treatment protocols and healthcare facilities may have influenced the observed mortality rates. The higher mortality observed in this study may be attributed to the delayed presentation of patients, often in septicaemia, shock, and multi-organ failure. These critical conditions at the time of surgery likely contributed to poor postoperative outcomes despite surgical intervention. Early presentation to healthcare facilities and timely resuscitation may improve survival in such high-risk patients. The predominance of perforation peritonitis and superior mesenteric artery thrombosis with extensive bowel gangrene in our region, likely influenced by dietary habits, further explains the observed mortality rates. Further research involving larger and more diverse cohorts is needed to refine our understanding of mortality rates and identify additional contributing factors.

Among the three patients who underwent elective procedures, one patient who underwent distal pancreatectomy for pancreatic cystadenoma died, with the cause of death attributed to septic shock secondary to a lower respiratory tract infection. The other two patients, who underwent a Whipple procedure for carcinoma of the pancreas and exploratory laparotomy with resection and end ileostomy for gastrointestinal stromal tumour, had uneventful recoveries. This highlights that while elective surgeries generally have better outcomes, postoperative infections can still lead to significant morbidity and mortality.

The study identified key causes of extubation failure among postoperative general surgery patients requiring mechanical ventilation. Ventilator-associated pneumonia and multiple organ dysfunction syndrome were significantly associated with extubation failure, contributing to prolonged ventilation and impaired recovery. Elevated procalcitonin levels indicated persistent systemic inflammation and infection, compromising respiratory function. High qSOFA score ≥ 2 and APACHE II scores reflected prolonged intubation, which increased the risk of respiratory muscle disuse atrophy and airway complications. Early identification of these factors can guide targeted interventions to improve outcomes, including infection management and tailored weaning protocols.

The study's prospective design strengthened its ability to establish temporal relationships, reduce recall bias, and provide a more reliable assessment of associations between exposures and outcomes. Limitations include the single-centre setting, relatively small sample size, absence of a control group, and lack of multivariate analysis, which may limit generalisability and the ability to adjust for confounders.

## Conclusions

Our study underscores the importance of clinical biomarkers and scoring systems, including qSOFA, APACHE II, INR, albumin, and procalcitonin in predicting mortality and guiding management strategies for general surgery patients requiring mechanical ventilation. Early risk stratification using these predictors can optimise decision-making, resource allocation, intervention strategies, and the counselling of patient attendees by providing prognostication in critically ill patients. Further research should aim to validate these findings and explore additional variables that could impact patient outcomes in critical care.
